# Estimating age-based antiretroviral therapy costs for HIV-infected children in resource-limited settings based on World Health Organization weight-based dosing recommendations

**DOI:** 10.1186/1472-6963-14-201

**Published:** 2014-05-02

**Authors:** Kathleen Doherty, Shaffiq Essajee, Martina Penazzato, Charles Holmes, Stephen Resch, Andrea Ciaranello

**Affiliations:** 1 Medical Practice Evaluation Center, Divisions of General Medicine, Massachusetts General Hospital, Boston, MA, USA; 2 Department of Pediatrics, New York University School of Medicine, New York, NY, USA; 3 Clinton Health Access Initiative, Boston, MA, USA; 4 Clinical Trial Unit Medical Research Council, London, UK; 5 Center for Infectious Disease Research in Zambia (CIDRZ), Lusaka, Zambia; 6 Center for Health Decision Science, Harvard School of Public Health, Boston, MA, USA; 7 Medical Practice Evaluation Center, Divisions of General Medicine and Infectious Disease, Massachusetts General Hospital, Boston, MA, USA

**Keywords:** Antiretroviral therapy, Pediatric HIV, Costs

## Abstract

**Background:**

Pediatric antiretroviral therapy (ART) has been shown to substantially reduce morbidity and mortality in HIV-infected infants and children. To accurately project program costs, analysts need accurate estimations of antiretroviral drug (ARV) costs for children. However, the costing of pediatric antiretroviral therapy is complicated by weight-based dosing recommendations which change as children grow.

**Methods:**

We developed a step-by-step methodology for estimating the cost of pediatric ARV regimens for children ages 0–13 years old. The costing approach incorporates weight-based dosing recommendations to provide estimated ARV doses throughout childhood development. Published unit drug costs are then used to calculate average monthly drug costs. We compared our derived monthly ARV costs to published estimates to assess the accuracy of our methodology.

**Results:**

The estimates of monthly ARV costs are provided for six commonly used first-line pediatric ARV regimens, considering three possible care scenarios. The costs derived in our analysis for children were fairly comparable to or slightly higher than available published ARV drug or regimen estimates.

**Conclusions:**

The methodology described here can be used to provide an accurate estimation of pediatric ARV regimen costs for cost-effectiveness analysts to project the optimum packages of care for HIV-infected children, as well as for program administrators and budget analysts who wish to assess the feasibility of increasing pediatric ART availability in constrained budget environments.

## Background

Currently, 3.4 million children are living with HIV throughout the world [1]. Early antiretroviral therapy (ART) has been shown to reduce morbidity and mortality by 75% in infants infected with HIV [2], yet only approximately 28% of HIV-infected children in need of treatment are estimated to be receiving ART [3]. Massive scale-up of pediatric HIV treatment is needed to prevent mortality and control the pediatric HIV epidemic, at a time when resources for HIV care are increasingly constrained [4].

To inform the scale-up of pediatric HIV care in the context of budget constraints, policy makers need accurate projections of antiretroviral drug (ARV) costs and a clear analysis of the cost-effectiveness of alternative ART strategies [5-8]. There are four key issues in the estimation of ART costs for children. First, specific medications, such as lopinavir/ritonavir (LPV/r) and abacavir (ABC), may be preferred in guidelines for clinical reasons [9-12], but their costs remain higher as well. Second, fixed-dose combinations (FDCs) are widely used in many ART programs and often lead to substantial cost-savings compared to their individual component drugs, as well as dosing convenience and improved adherence [13-15]. The production of pediatric FDCs has improved drastically over the past few years, resulting in several dual and triple dispersible formulations [7,16]. However, access to these formulations remains limited in many programs [17,18]. Third, many medications are not formulated for easy administration to infants and very young children, who cannot yet swallow pills. Such children require either dispersible tablets or syrup formulations, which are often more expensive than tablets of the same medication, and in the case of syrups may be less palatable, compromising adherence and efficacy [19]. Finally, national and international recommendations regarding pediatric ART treatment are in transition. Although the WHO publishes guidelines on pediatric HIV treatment, differences in first and second line treatment regimens exist between Asian and African care settings [20]. Additionally, discrepancies between preferred regimens exist within individual countries between different programs or clinics. In general, programs recommend that children are transitioned away from syrups to solid formulations as soon as they are able to tolerate them. This reduces costs, but local clinical practice may differ from the national recommendation.

Pediatric antiretroviral therapy use is further complicated by the need for increasing drug doses as children grow. Patients must be consistently monitored for changes in weight to ensure that they are receiving adequate doses of each drug to reach therapeutic levels and fully suppress the virus, while also avoiding toxicities related to excess dosing [21]. To evaluate the costs and cost-effectiveness of various ARV regimens analysts need a defined methodology for calculating monthly ARV costs that incorporates this need for dose modification, but a simple method to estimate weight-based costs is not widely available. In 2005, the Clinton Health Access Initiative (CHAI) developed a forecasting tool for pediatric ART which has primarily been used to help countries determine the cost of pediatric treatment targets and as a tool for ARV procurement planning. The tool estimates the quantities of ARVs needed based on the makeup of the patient population and then allows program managers to input current drug pricing to create a full ARV program budget. However, CHAI’s model has not been published to date. Two published studies have also examined pediatric HIV treatment costs. One reports the average cost of HIV treatment care across 43 PEPFAR sites, where 7% of the study population were pediatric patients [22]. Meyer-Rath *et al*. completed a similar analysis for two dedicated pediatric care sites in South Africa, and further calculated the proportion of total treatment costs pertaining to ART costs [23]. However, neither study broke down the cost of pediatric ART by age, weight or regimen.

Here, we propose a step-by-step approach for determining the monthly costs of ARV medications for children ages 0–13 years in resource-limited settings. We use published ARV price lists as a basis, and account for changes in dosing as children grow, as well as the possible lack of availability of pediatric tablets or co-formulations in some settings and for some groups of patients. This methodology can be easily updated using a simple online tool as new ARV prices are released by international agencies [24,25] or are negotiated by individual governments [26,27] (see Additional file 1). We hope this approach can be used by investigators seeking to analyze the cost-effectiveness of ART strategies for children, as well as by analysts and program administrators in accurately assessing the costs of potential treatment options for pediatric HIV patients.

## Methods

To estimate the monthly cost of specific ART regimens for children ages 0–13 years in resource-limited settings, we developed a 7-step approach.

1. *Weight-based doses for* pediatric ARV were taken from Annex E of the 2010 World Health Organization (WHO) Pediatric Guidelines and Annex 7 of the upcoming WHO 2013 Consolidated Pediatric and Adult Guidelines (Table 1) [7,28]. The WHO stratifies dosing recommendations by drug formulation and by current weight (divided into 7 weight bands: 3–5.9 kg, 6–9.9 kg, 10–13.9 kg, 14–19.9 kg, 20–24.9 kg, 25–34.9 kg, and 35+ kg). We modeled dosing for all drugs that are currently recommended in the 2013 WHO guidelines. We also included doses from the 2010 WHO guidelines for drugs such as stavudine (d4T) that are no longer recommended, but still likely to be available in many pediatric treatment programs [7].

2. *Assignment of unit costs for each antiretroviral drug (Table *2*).* We next identified publicly available unit costs for each medication. Unit costs are defined as cost (in 2012 USD) per tablet, per capsule or per milliliter of liquid formulation. Due to the wide variety of drug prices available to resource-limited countries based on individual agreements with pharmaceutical companies, we used the CHAI May 2012 ceiling price list as the basis for drug cost estimates [24]. These ceiling prices represent the highest level that pharmaceutical companies can charge for these brand-name products in the countries that are members of the CHAI Procurement Consortium, and thus that have access to the reduced costs drug costs negotiated by CHAI. As individual countries or programs may be able to negotiate or obtain access to lower prices for certain formulations, the unit costs shown here should be taken as a maximum estimate of drug costs in resource-limited settings. For the formulations not included in the CHAI 2012 price list, we used the price list from Mèdecins Sans Frontières (MSF), and chose the price estimate for the generic version of the drug formulation if available [25]. Any other price list could be substituted for this source of data, including prices negotiated by other international agencies or national governments [29]. This analysis includes only the costs of drug products themselves, and excludes the costs of shipping, or any costs related to the delivery of ART, such as equipment, personnel, overhead, or wasting.

3. *Calculation of monthly costs for each medication by weight (Table *3*).* Assuming an average of 30.4 days in a month, we calculate the monthly cost for each medication by WHO weight band as follows:

$$\begin{array}{l} \mathrm{Monthly}\;\mathrm{cost}=\mathrm{Average}\;\mathrm{dose}\;\mathrm{per}\;\mathrm{day}*\\ \;\mathrm{unit}\;\mathrm{cost}\;\mathrm{per}\;\mathrm{drug}*\\ \;\mathrm{30.4}\;\mathrm{days}\;\mathrm{per}\;\mathrm{month} \end{array}$$

Average daily dose (in mg, ml, tablet, or capsule/day) is found in Table 2 and unit cost (in USD/mg, ml, tablet, or capsule) is found is Table 3 for each of the drugs in this analysis.

4. *Probability of being in each WHO weight band by age (Table *4*).* Although most clinical guidelines regarding ARV dosing recommendations are based on a child’s weight, rather than current age [17], most policy recommendations, such as the WHO criteria for ART initiation, are based on a child’s age at presentation to care [7]. Thus, it is useful to translate the weight-based ARV costs calculated in Step 3 (Table 3) into age-based ARV costs. Weight-by-age distributions were obtained from the WHO Child Growth Standards for children <5 years of age and from the Centers for Disease Control and Prevention (CDC) Growth Charts for children ≥5 years [30,31]. Both growth charts contain the Lambda-Mu-Sigma (LMS) parameters (the power in the Box-Cox transformation (L), the median (M), and the generalized coefficient of variation (S)) needed to generate exact percentiles and z-scores by age and gender [32].

Although few data are available on how the weight distribution of HIV-infected children compares to the weight distribution of the general population of children the same age, one study found that on average, HIV-infected children are of lower weight [33]. To account for lower weights among HIV-infected children than among the general population, we first shifted the weight distribution in the growth charts to a -1.5 z-score (Z) and assumed the coefficient of variation of the weight distribution (S_HIV_) was equal to that of the general population. To examine the impact of these assumptions, we conducted sensitivity analyses in which we recalculated drug costs after varying both the shifted weight distribution z-score (z-scores of -3 to 0) and the coefficient of variation (double and half S_HIV_). These parameters can also be modified by use of the online tool.

Using these new weight-by age distributions, we calculated Z-scores for the upper and lower bounds of the weight bands used in the WHO dosing recommendations (3 kg, 5.9 kg, 6 kg, 9.9 kg, 10 kg, 13.9 kg, 14 kg, 19.9 kg, 20 kg, 24.9 kg, 25 kg, and 34.9 kg). An automated calculation function in Excel was then used to convert the z-scores into cumulative probability estimates. Finally, the probability of being in each weight band (by age and gender) was calculated as the cumulative probability of being above the upper bound of the weight band subtracted from the cumulative probability of being below the lower bound of the weight band (or the area under the curve within the upper and lower bounds). Assuming an equal proportion of males and females, these final probabilities by gender were averaged to estimate the probability of being in each weight band by age (Table 4).

5. *Calculation of monthly costs for each medication by age (Table*5*).* We then used the newly defined probabilities of being in each weight band by age (Table 4) to calculate monthly medication costs by age. Monthly medication costs by age were calculated as the monthly medication cost by weight band (Table 3) multiplied by the probability of being in each weight band (Table 4). Several ARVs (such as ABC) have more than one formulation option recommended at each age. For these medications, we provide estimated monthly costs for two formulation options: 1) children initiate pediatric liquid formulations and then switch to pediatric then adult tablet formulations once liquids are no longer recommended or 2) children begin pediatric tablet formulations as soon as recommended, and then switch to adult tablet formulations once pediatric tablets are no longer recommended.

6. *Calculation of monthly costs for complete medication regimens (Table*6*).* To determine the monthly costs of complete ART regimens, we combined the monthly costs by age for each of the component medications. The WHO 2013 HIV treatment guidelines currently recommend a first-line, PI-based regimen (LPV/r + 3TC + ABC or ZDV) for all HIV-infected children less than three years of age. However, previous WHO guidelines had recommended an NNRTI-based regimen for children who had not been exposed to NNRTIs to prevent mother-to-child transmissions, and in many countries, NNRTI-based regimens remain the most readily available first-line option for children. Here, we present examples of monthly regimen cost calculations for six commonly used ART regimens: lopinavir/ritonavir (LPV/r) with zidovudine (ZDV) and lamivudine (3TC), LPV/r with abacavir (ABC) and 3TC, LPV/r with stauvudine (d4T) and 3TC, nevirapine (NVP) or efavirenz (EFV) with ABC and 3TC, NVP or EFV with ZDV and 3TC, and NVP or EFV with d4T and 3TC. Although d4T is no longer recommended as a first-line medication, it remains an option to use in case of anemia where access to ABC is limited; d4T is commonly used throughout Africa, largely because of its availability in inexpensive FDC formulations [7,35].

For each regimen, we derived costs for three key scenarios that reflect setting-specific variations in the availability of co-formulated FDCs and the age at which children are assumed to transition from liquid to tablet formulations:

**Scenario 1 (No FDCs, Late Switch):** Coformulations are not available. Liquid formulations of all drug components are used until 4 years of age, and then children switch to pediatric/adult tablets as recommended by weight-based dosing.

**Scenario 2 (No FDCs, Early Switch):** Coformulations are not available. Liquid formulations of all drug components are used until 6 months of age, and then children switch to pediatric/adult tablets as recommended by weight-based dosing.

**Scenario 3 (Available FDCs, Early Switch):** Coformulations are available. Coformulated pediatric tablets, if they exist, are used from birth. If all drug components are not included in the coformulation, liquid formulations of the remaining drugs are used until 6 months of age, and then children switch to pediatric/adult tablets as recommended by weight-based dosing.

For example, South Africa currently has access to most pediatric liquid and tablet formulations, but co-formulations are not widely available [17,18]. Therefore, when compiling monthly regimen costs for South Africa, individual liquid or tablet doses must be used instead of the fixed dose combination pills (Scenarios 1 and 2). The above scenarios reflect only three possible assumptions regarding the settings for pediatric ART availability. Alternate scenarios can be analyzed with use of the online tool.

7. *Comparison of derived to published costs.* After completion of the six steps above, we compared our calculated monthly ART costs (by weight) to other published costs. The WHO has compiled a Global Price Reporting Mechanism (GPRM) database, which contains average yearly costs for both adult and pediatric ARVs, based on international transactions of HIV, tuberculosis and malaria medications purchased in resource-limited settings [36]. Costs for pediatric drug formulations in the GPRM database are shown only for children weighing 10kg. Here, we compare both pediatric and adult GPRM costs to the costs derived for the most relevant weight band in our analysis (pediatric: weight bands 6–9.9 kg and 10–13.9 kg adult: 35+ kg).

**Table 1:** **Weight-based dosing recommendations for currently recommended and commonly** used antiretroviral drugs [7,28]

	**WHO dosing recommendations by weight band**						
**Medication (strength)**	**3-5.9 kg**	**6-9.9 kg**	**10-13.9 kg**	**14-19.9 kg**	**20-24.9 kg**	**25-34.9 kg**	**35+ kg**
ABC (20 mg/ml)	3 ml bid	4 ml bid	6ml bid	-	-	-	-
ABC (60 mg)	1 tab bid	1.5 tab bid	2 tab bid	2.5 tab bid	3 tab bid	-	-
ABC (300 mg)	-	-	-	-	-	1 tab bid	1 tab bid
d4T (1 mg/ml)	6 ml bid	9 ml bid	-	-	-	-	-
d4T (15 mg)	-	-	1 tab bid	-	-	-	-
d4T (20 mg)	-	-	-	1 tab bid	1 tab bid	-	-
d4T (30 mg)	-	-	-	-	-	1 tab bid	1 tab bid
3TC (10 mg/ml)	3 ml bid	4 ml bid	6 ml bid	-	-	-	-
3TC (150 mg)	-	-	-	0.5 tab bid	1 tab am + 0.5 tab pm	1 tab bid	1 tab bid
EFV (200 mg)	NR	NR	1 cap qd (≥3yrs only)	1.5 cap qd	1.5 cap qd	2 cap qd	-
EFV (600 mg)	-	-	-	-	-	-	1 tab qd
LPV/r (80/20 mg/ml)	1 ml bid	1.5 ml bid	2ml bid	2.5 ml bid	3 ml bid	-	-
LPV/r (100/25 mg)	NR	NR	2 tab am + 1 tab pm	2 tab bid	2 tab bid	3 tab bid	-
LPV/r (200/50 mg)	-	-	-	-	-	-	2 tab bid
NVP (10 mg/ml)	5 ml bid	8ml bid	10 ml bid	-	-	-	-
NVP (50 mg)	1 tab bid	1.5 tab bid	2 tab bid	2.5 tab bid	3 tab bid	-	-
NVP (200 mg)	-	-	-	-	-	1 tab bid	1 tab bid
ZDV (10 mg/ml)	6 ml bid	9 ml bid	12 ml bid	-	-	-	-
ZDV (60 mg)	1 tab bid	1.5 tab bid	2 tab bid	2.5 tab bid	3 tab bid	-	-
ZDV (300 mg)	-	-	-	-	-	1 tab bid	1 tab bid
ZDV/3TC^A^ (60 mg/30 mg)	1 tab bid	1.5 tab bid	2 tab bid	2.5 tab bid	3 tab bid	-	-
ZDV/3TC^A^ (300 mg/150 mg)	-	-	-	-	-	1 tab bid	1 tab bid
ZDV/3TC/NVP^B^ (60 mg/30 mg/50 mg)	1 tab bid	1.5 tab bid	2 tab bid	2.5 tab bid	3 tab bid	-	-
ZDV/3TC/NVP^B^ (300 mg/150 mg/200 mg)	-	-	-	-	-	1 tab bid	1 tab bid
d4T/3TC (6 mg/30 mg)^C^	1 tab bid	1.5 tab bid	2 tab bid	2.5 tab bid	3 tab bid	-	-
d4T/3TC^C^ (30 mg/150 mg)	-	-	-	-	-	1 tab bid	1 tab bid
d4T/3TC/NVP^D^ (6 mg/30 mg/50 mg)	1 tab bid	1.5 tab bid	2 tab bid	2.5 tab bid	3 tab bid	-	-
d4T/3TC/NVP^D^ (30 mg/150 mg/200 mg)	-	-	-	-	-	1 tab bid	1 tab bid
ABC/3TC^E^ (60 mg/30 mg)	1 tab bid	1.5 tab bid	2 tab bid	2.5 tab bid	3 tab bid	-	-
ABC/3TC^E^ (600 mg/300 mg)	-	-	-	-	-	0.5 tab bid	0.5 tab bid

*Abbreviations*: ***ABC*** abacavir, ***d4T*** stavudine, ***3TC*** lamivudine, ***EFV*** efavirenz, ***LPV/r*** lopinavir/ritonavir, ***NVP*** nevirapine, ***ZDV*** zidovudine, ***mg*** milligrams, ***ml*** milliliter, ***bid*** twice daily, ***qd*** once daily, ***NR*** not recommended, ***tab*** tablet, ***cap*** capsule, ***kg*** kilogram.

A. Pediatric tablet is scored and can be split. Can be crushed and contents mixed with a small amount of water or food and taken immediately.

B. Pediatric tablet is dispersible and may be split. Can be dispersed into a small volume of water and mixed into a small amount of food and taken immediately.

C. Pediatric tablet is dispersible and crushable, can be split.

D. Pediatric tablet is dispersible and crushable, can be split. If unable to swallow, disperse 1 tablet in 2 teaspoons of water.

E. Pediatric tablet is scored and can be split. Can be crushed and contents mixed with a small amount of water or food and taken immediately.

**Table 2:** **Unit costs, derived from CHAI 2012 Price List**[24]

**Medication (strength)**	**Unit cost (2012 USD)**
ABC (20 mg/ml)	$0.06/ml
ABC (60 mg)	$0.09/tab
ABC (300 mg)	$0.26/tab
d4T (1 mg/ml)	$0.01/tab
d4T (15 mg)	$0.02/tab
d4T (20 mg)	$0.02/tab
d4T (30 mg)	$0.03/tab
3TC (10 mg/ml)	$0.01/ml
3TC (150 mg)	$0.04/tab
EFV (200 mg)	$0.11/cap
EFV (600 mg)	$0.15/tab
LPV/r (80/20 mg/ml)	$0.19/ml
LPV/r (100/25 mg)	$0.14/tab
LPV/r (200/50 mg)	$0.26/tab
NVP (10 mg/ml)	$0.01/ml
NVP (50 mg)	$0.05/tab^A^
NVP (200 mg)	$0.05/tab
ZDV (10 mg/ml)	$0.01/ml
ZDV (60 mg)	$0.05/tab^B^
ZDV (300 mg)	$0.12/tab
ZDV/3TC (60 mg/30 mg)	$0.05/tab
ZDV/3TC (300 mg/150 mg)	$0.15/tab
ZDV/3TC/NVP (60 mg/30 mg/50 mg)	$0.07/tab
ZDV/3TC/NVP (300 mg/150 mg/200 mg)	$0.19/tab
d4T/3TC (6 mg/30 mg)	$0.03/tab
d4T/3TC (30 mg/150 mg)	$0.06/tab
d4T/3TC/NVP (6 mg/30 mg/50 mg)	$0.04/tab
d4T/3TC/NVP (30 mg/150 mg/200 mg)	$0.11/tab
ABC/3TC (60 mg/30 mg)	$0.09/tab
ABC/3TC (600 mg/300 mg)	$0.58-$1.07/tab^C^

*Abbreviations*: ***ABC*** abacavir, ***d4T*** stavudine, ***3TC*** lamivudine, ***EFV*** efavirenz, ***LPV/r*** lopinavir/ritonavir, ***NVP*** nevirapine, ***ZDV*** zidovudine, *** mg*** milligrams, ***ml*** milliliter, ***tab*** tablet, ***cap*** capsule.

**A**. Cost for NVP (50 mg) not reported in CHAI Price List. Mèdecins San Frontières (MSF) reports a cost of $0.05/tab from manufacturer Aurobindo [25].

**B**. Cost for ZDV (60 mg) not reported in 2012 CHAI Price List. MSF reports a cost of $0.05/tab from manufacturer Ranbaxy [25].

**C**. Cost for ABC/3TC (600 mg/300 mg) not reported in 2012 CHAI Price List. MSF reports a cost of $0.58-$1.07/tab from manufacturers ViiV, Aurobindo, Cipla and Matrix [25].

**Table 3:** Monthly costs for key antiretroviral drugs by weight

	**Weight bands**						
**Medication (strength)**	**3-5.9 kg**	**6-9.9 kg**	**10-13.9 kg**	**14-19.9 kg**	**20-24.9 kg**	**25-34.9 kg**	**35+ kg**
ABC (20 mg/ml)	$10.94	$14.59	$21.89	-	-	-	-
ABC (60 mg)	$5.47	$8.21	$10.94	$13.68	$16.42	-	-
ABC (300 mg)	-	-	-	-	-	$15.81	$15.81
d4T (1 mg/ml)	$3.65	$5.47	-	-	-	-	-
d4T (15 mg)	-	-	$1.22	-	-	-	-
d4T (20 mg)	-	-	-	$1.22	$1.22	-	-
d4T (30 mg)	-	-	-	-	-	$1.82	$1.82
3TC (10 mg/ml)	$1.82	$2.43	$3.65	-	-	-	-
3TC (150 mg)	-	-	-	$1.22	$1.82	$2.43	$2.43
EFV (200 mg)	NR	NR	$3.34	$5.02	$5.02	$6.69	-
EFV (600 mg)	-	-	-	-	-	-	$4.56
LPV/r (80/20 mg/ml)	$11.55	$17.33	$23.10	$28.88	$34.66	-	-
LPV/r (100/25 mg)	NR	NR	$12.77	$17.02	$17.02	$25.54	-
LPV/r (200/50 mg)	-	-	-	-	-	-	$31.62
NVP (10 mg/ml)	$3.04	$4.86	$6.08	-	-	-	-
NVP (50 mg)	$2.74	$4.10	$5.47	$6.84	$8.21	-	-
NVP (200 mg)	-	-	-	-	-	$3.04	$3.04
ZDV (10 mg/ml)	$3.65	$5.47	$7.30	-	-	-	-
ZDV (60 mg)	$3.04	$4.56	$6.08	$7.60	$9.12	-	-
ZDV (300 mg)	-	-	-	-	-	$7.30	$7.30
ZDV/3TC (60 mg/30 mg)	$3.04	$4.56	$6.08	$7.60	$9.12	-	-
ZDV/3TC (300 mg/150 mg)	-	-	-	-	-	$9.12	$9.12
ZDV/3TC/NVP (60 mg/30 mg/50 mg)	$4.26	$6.38	$8.51	$10.64	$12.77	-	-
ZDV/3TC/NVP (300 mg/150 mg/200 mg)	-	-	-	-	-	$11.55	$11.55
d4T/3TC (6 mg/30 mg)	$1.82	$2.74	$3.65	$4.56	$5.47	-	-
d4T/3TC (30 mg/150 mg)	-	-	-	-	-	$3.65	$3.65
d4T/3TC/NVP (6 mg/30 mg/50 mg)	$2.43	$3.65	$4.86	$6.08	$7.30	-	-
d4T/3TC/NVP (30 mg/150 mg/200 mg)	-	-	-	-	-	$6.69	$6.69
ABC/3TC (60 mg/30 mg)	$5.47	$8.21	$10.94	$13.68	$16.42	-	-
ABC/3TC (600 mg/300 mg)	-	-	-	-	-	$17.72-$32.53	$17.72-$32.53

*Abbreviations*: ***ABC*** abacavir, ***d4T*** stavudine, ***3TC*** lamivudine, ***EFV*** efavirenz, ***LPV/r*** lopinavir/ritonavir, **NVP** nevirapine, ***ZDV*** zidovudine, ***mg*** milligrams, ***ml*** milliliter, ***NR*** not recommended, ***kg*** kilogram.

**Table 4:** Probability of being in each WHO weight band by age

	**Weight bands**						
**Age (years)**	**3-5.9 kg**	**6-9.9 kg**	**10-13.9 kg**	**14-19.9 kg**	**20-24.9 kg**	**25-34.9 kg**	**35+ kg**
0.5	0.246	0.754	0.000	0.000	0.000	0.000	0.000
1	0.001	0.908	0.091	0.000	0.000	0.000	0.000
2	0.000	0.285	0.706	0.009	0.000	0.000	0.000
3	0.000	0.044	0.842	0.113	0.000	0.000	0.000
4	0.000	0.003	0.593	0.398	0.005	0.000	0.000
5	0.000	0.000	0.262	0.696	0.039	0.003	0.000
6	0.000	0.000	0.070	0.779	0.135	0.016	0.001
7	0.000	0.000	0.011	0.638	0.292	0.057	0.003
8	0.000	0.000	0.001	0.403	0.439	0.147	0.010
9	0.000	0.000	0.000	0.197	0.481	0.293	0.029
10	0.000	0.000	0.000	0.073	0.390	0.464	0.073
11	0.000	0.000	0.000	0.019	0.234	0.586	0.162
12	0.000	0.000	0.000	0.003	0.096	0.591	0.310
13	0.000	0.000	0.000	0.000	0.023	0.465	0.512

*Abbreviation*: ***kg*** kilogram.

Weight-by-age standards were tabulated from the WHO Child Growth Standards for children <5 years and from the CDC Growth Charts for children ages ≥5 years [30,31]. The equations used to generate these age-specific weight distributions rely on the Lambda-Mu-Sigma parameters: the median (M) weight, the generalized coefficient of variation (S), and the power in the Box-Cox transformation (L) [32,34]. To account for lower weights among HIV-infected children relative to the general population of same age, the age-specific weight distributions for HIV-infected children were shifted to the -1.5 z-score of the general population distributions [33]. The HIV-infected median weight-for-age values (M_HIV_) were calculated at half-year intervals, where M_HIV_ was equal to the value M*(1 + LSZ)^(1/L)^. The coefficient of variation (S_HIV_) was assumed to be equal to that of children of the same age in the general population. Z-scores for the upper and lower bounds of the WHO-specified weight intervals for ARV dosing (3 kg, 5.9 kg, 6 kg, 9.9 kg, 10 kg, 13.9 kg, 14 kg, 19.9 kg, 20 kg, 24.9 kg, 25 kg, and 34.9 kg) were calculated by gender for children ages 0–13 years old, as follows: the z-score of the bound of interest, X, equals ((X/M_HIV_)^L^ -1)/L*S_HIV_. The Norm.S.Dist(z,1) Excel function was used to convert z-scores into cumulative probability estimates. The probability of being in each weight band (by age and gender) was calculated as the cumulative probability of being below the upper bound of the weight band subtracted by the cumulative probability of being below the lower bound of the weight band (or the area under the curve within the upper and lower bounds). Results shown above assume an equal proportion of males and females.

**Table 5:** Monthly costs for antiretroviral drugs by age

	**Age (years)**													
**Drug product**	**0.5**	**1**	**2**	**3**	**4**	**5**	**6**	**7**	**8**	**9**	**10**	**11**	**12**	**13**
**(Formulation)**														
ABC^A^	$13.70	$15.26	$19.74	$20.63	$18.57	$15.94	$14.65	$14.70	$15.23	$15.68	$15.89	$15.91	$15.86	$15.82
ABC^B^	$7.54	$8.46	$10.19	$11.13	$12.06	$13.08	$13.89	$14.57	$15.21	$15.68	$15.89	$15.91	$15.86	$15.82
d4T	$5.02	$5.08	$2.43	$1.40	$1.23	$1.22	$1.23	$1.25	$1.31	$1.41	$1.54	$1.67	$1.76	$1.81
3TC	$2.28	$2.54	$3.28	$3.32	$2.67	$1.88	$1.49	$1.49	$1.68	$1.90	$2.11	$2.27	$2.37	$2.42
EFV	NR	NR	NR	$3.39	$4.01	$4.58	$4.93	$5.09	$5.26	$5.49	$5.76	$5.92	$5.86	$5.56
LPV/r^A^	$15.91	$17.85	$21.51	$23.51	$25.45	$27.58	$29.21	$30.32	$30.94	$30.75	$29.78	$28.71	$28.31	$28.86
LPV/r^B^	$15.91	$16.91	$14.11	$13.45	$14.50	$15.93	$16.87	$17.50	$18.41	$19.94	$22.04	$24.37	$26.58	$28.46
NVP^A^	$4.42	$4.97	$5.74	$6.11	$6.39	$6.68	$6.91	$7.00	$6.84	$6.27	$5.33	$4.32	$3.55	$3.16
NVP^B^	$3.77	$4.23	$5.09	$5.57	$6.03	$6.52	$6.87	$7.00	$6.84	$6.27	$5.33	$4.32	$3.55	$3.16
ZDV^A^	$5.02	$5.64	$6.78	$7.25	$7.42	$7.58	$7.78	$8.02	$8.22	$8.23	$8.03	$7.73	$7.47	$7.34
ZDV^B^	$4.19	$4.70	$5.66	$6.19	$6.70	$7.26	$7.69	$8.01	$8.22	$8.23	$8.03	$7.73	$7.47	$7.34
ZDV/3TC	$4.19	$4.70	$5.66	$6.19	$6.70	$7.27	$7.72	$8.12	$8.50	$8.82	$9.01	$9.09	$9.12	$9.12
ZDV/3TC/NVP	$5.86	$6.58	$7.93	$8.66	$9.38	$10.17	$10.79	$11.29	$11.71	$11.96	$11.96	$11.82	$11.67	$11.58
d4T/3TC	$2.51	$2.82	$3.40	$3.71	$4.02	$4.35	$4.61	$4.76	$4.82	$4.70	$4.43	$4.09	$3.83	$3.69
d4T/3TC/NVP	$3.35	$3.76	$4.53	$4.95	$5.36	$5.81	$6.17	$6.46	$6.71	$6.86	$6.88	$6.82	$6.74	$6.70
ABC/3TC	$7.54	$8.46	$10.19	$11.13	$12.06	$13.08	$13.92	$14.69	$15.51	$16.30	$16.92	$17.34	$17.59	$17.69

Abbreviations: ***ABC*** abacavir, ***d4T*** stavudine, ***3TC*** lamivudine, ***EFV*** efavirenz, ***LPV/r*** lopinavir/ritonavir. ***NVP*** nevirapine, ***ZDV*** zidovudine, ***NR*** not recommended.

A. Patients are assumed to start the liquid formulation of the medication and then switch directly to pediatric or adult tablets when recommended (by weight).

B. Patients are assumed to start pediatric tablet formulations of the medication at 0.5 years old (or at earliest recommended age) and then switch to adult tablets when recommended (by weight).

**Table 6:** Monthly regimen costs: Six commonly used regimens

	**Age (years)**													
**Regimen**^**A**^	**0.5**	**1**	**2**	**3**	**4**	**5**	**6**	**7**	**8**	**9**	**10**	**11**	**12**	**13**
**LPV/R + ZDV + 3TC**														
Scenario 1	$23.22	$26.03	$31.57	$34.08	$23.86	$25.07	$26.05	$27.00	$28.30	$30.07	$32.17	$34.36	$36.42	$38.21
Scenario 2	$22.38	$24.15	$23.05	$22.96	$23.86	$25.07	$26.05	$27.00	$28.30	$30.07	$32.17	$34.36	$36.42	$38.21
Scenario 3	$20.10	$21.61	$19.77	$19.64	$21.20	$23.20	$24.59	$25.61	$26.91	$28.76	$31.04	$33.46	$35.69	$37.58
**LPV/R + ABC + 3TC**														
Scenario 1	$31.89	$35.65	$44.53	$47.46	$29.22	$30.89	$32.25	$33.56	$35.29	$37.52	$40.03	$42.55	$44.81	$46.70
Scenario 2	$25.73	$27.91	$27.57	$27.91	$29.22	$30.89	$32.25	$33.56	$35.29	$37.52	$40.03	$42.55	$44.81	$46.70
Scenario 3	$23.45	$25.36	$24.30	$24.59	$26.56	$29.01	$30.79	$32.18	$33.92	$36.23	$38.95	$41.71	$44.16	$46.15
**LPV/R + D4T +3TC**														
Scenario 1	$23.22	$25.48	$27.22	$28.23	$18.40	$19.03	$19.58	$20.24	$21.40	$23.25	$25.68	$28.31	$30.71	$32.68
Scenario 2	$23.21	$24.53	$19.81	$18.17	$18.40	$19.03	$19.58	$20.24	$21.40	$23.25	$25.68	$28.31	$30.71	$32.68
Scenario 3	$18.42	$19.73	$17.50	$17.16	$18.52	$20.29	$21.47	$22.26	$23.22	$24.64	$26.46	$28.46	$30.40	$32.15
**NVP OR EFV + ZDV + 3TC**^**B**^														
Scenario 1	$11.72	$13.15	$15.80	$13.96	$13.37	$13.72	$14.11	$14.59	$15.15	$15.63	$15.89	$15.92	$15.71	$15.32
Scenario 2	$10.24	$11.47	$14.04	$12.89	$13.37	$13.72	$14.11	$14.59	$15.15	$15.63	$15.89	$15.92	$15.71	$15.32
Scenario 3	$5.86	$6.58	$7.93	$9.57	$10.71	$11.85	$12.65	$13.21	$13.76	$14.31	$14.77	$15.01	$14.98	$14.68
**NVP OR EFV + ABC + 3TC**^**B**^														
Scenario 1	$20.40	$22.77	$28.76	$27.34	$18.73	$19.54	$20.31	$21.16	$22.14	$23.07	$23.75	$24.10	$24.09	$23.80
Scenario 2	$13.59	$15.23	$18.56	$17.84	$18.73	$19.54	$20.31	$21.16	$22.14	$23.07	$23.75	$24.10	$24.09	$23.80
Scenario 3	$11.31	$12.68	$15.28	$14.52	$16.06	$17.66	$18.85	$19.78	$20.76	$21.79	$22.68	$23.26	$23.45	$23.25
**NVP OR EFV + D4T +3TC**^**B**^														
Scenario 1	$11.72	$12.60	$11.45	$8.11	$7.90	$7.68	$7.64	$7.84	$8.24	$8.81	$9.41	$9.86	$10.00	$9.79
Scenario 2	$11.07	$11.85	$10.80	$8.11	$7.90	$7.68	$7.64	$7.84	$8.24	$8.81	$9.41	$9.86	$10.00	$9.79
Scenario 3	$3.35	$3.76	$4.53	$7.10	$8.03	$8.94	$9.53	$9.85	$10.07	$10.20	$10.18	$10.01	$9.69	$9.25

*Abbreviations*: ***ABC*** abacavir, ***d4T*** stavudine, ***3TC*** lamivudine, ***EFV*** efavirenz, ***LPV/r*** lopinavir/ritonavir, ***NVP*** nevirapine, ***ZDV*** zidovudine,

***A***. ARV availability scenarios (see also Methods):

**Scenario 1 (No FDCs, Late Switch):** Coformulations are not available. Liquid formulations of all drug components are used until 4 years of age, and then children switch to pediatric/adult tablets as recommended by weight-based dosing.

**Scenario 2 (No FDCs, Early Switch):** Coformulations are not available. Liquid formulations of all drug components are used until 6 months of age, and then children switch to pediatric/adult tablets as recommended by weight-based dosing.

**Scenario 3 (Available FDCs, Early Switch):** Coformulations are available. Coformulated pediatric tablets, if they exist, are used from birth. If all drug components are not included in the coformulation, liquid formulations of the remaining drugs are used until 6 months of age, and then children switch to pediatric/adult tablets as recommended by weight-based dosing.

**B**. Patients on the non-nucleoside reverse transcriptase inhibitor (NNRTI)-based regimens are assumed to receive NVP until age 3, then EFV at age 3 and after because EFV is not yet approved for children less than 3 years of age or 10kg of weight.

## Results

1. *Weight-based doses for each antiretroviral drug (Table*1*).* Table 1 lists the recommended daily dose of each drug across all seven weight bands used in the WHO 2010 and 2013guidelines.

2. *Assignment of unit costs for each antiretroviral drug (Table *2*).* Unit costs for each drug, using CHAI and MSF costs as an example, are shown in Table 2[24,25].

3. *Calculation of monthly costs for each medication and weight band (Table*3*).* With daily dosing recommendations and unit costs by drug, we then calculated monthly costs for each drug, stratified by weight band. Monthly costs for each drug formulation are shown in Table 3 for each WHO weight band where that drug formulation is recommended.

4. *Probability of being in each WHO weight band by age (Table*4*).*The probability of being in each WHO weight band by current age and gender was calculated as described in the Methods. Results are shown in Table 4 assuming an equal distribution of males and females.

Sensitivity analyses on weight distributions demonstrated that monthly drug cost estimates were fairly robust to changes in assumptions regarding the weight distribution for HIV-infected children (z-scores). Assuming no change in weight distribution between HIV-infected children and the general population (z-score = 0), monthly drug costs by age changed on average by 9.7%. Monthly drug costs changed substantially from our original estimates (>10%) only when we assumed much lower weights among HIV-infected children (z-score ≤ -3). With improvements in early infant diagnosis and access to antiretroviral therapy (ART), we anticipate that the growth distribution for HIV-infected children will shift toward the WHO growth curves for the general population, and thus drug costs will remain similar to those calculated in this manuscript. Sensitivity analyses on the coefficient of variation of the weight distribution among HIV-infected children (S_HIV_) also demonstrated minimal change in monthly drug costs estimates. First, assuming that S_HIV_ was equal to half the coefficient of variation of the general population, we found that monthly drug costs changed by 4.2%, on average. Second, assuming S_HIV_ was double the coefficient of variation of the general population, we found that monthly drug costs changed on average by 6.4%.

5. *Calculation of monthly costs for each medication by age (Table*5*).* Using the probabilities of being in each weight band (Table 4) and the monthly costs for each medication by weight band (Table 3), we calculated the monthly cost for each medication by age (Table 5).

6. *Calculation of monthly costs for complete medication regimens (Table*6*).* Monthly costs for complete regimens are shown in Table 6, for a variety of regimens and formulations to reflect the range of settings described in the Methods. As seen in the Table, monthly regimen costs do not increase monotonically as a child grows. This is due to the switch from more expensive liquid and pediatric tablet formulations to adult tablet doses. In addition, the switch from NVP to EFV at age three also increases overall monthly regimen costs.

7. *Comparison of derived to published costs.* Compared to the pediatric drug costs reported in the WHO GPRM database (based on children weighing 10 kg), the costs derived in our analysis for children in weight bands 6–9.9 kg and 10–13.9 kg were fairly comparable or slightly higher for most formulations (Table 7). One exception of note is our estimate of LPV/r syrup costs (80/20 mg/ml). Using our methodology, the cost range for this formulation in weight bands 6–9.9 kg and 10–13.9 kg was almost double that reported in the GPRM ($17.33-$23.10 versus $11.81). All costs estimates from our analysis for the 35+ kg weight band were also comparable or slightly higher than the adult formulation costs reported in the WHO GPRM (Table 8).

**Table 7:** Comparison of pediatric formulation derived costs to the WHO Global Price Reporting Mechanism (GPRM) database

**Medication (strength)**	**Monthly costs for 6–9.9 kg**	**Monthly costs for 10–13.9 kg**	**Monthly costs**^**A**^
			**From GPRM**
	**(Table **3**)**	**(Table **3**)**	
ABC (20 mg/ml)	$14.59	$21.89	$15.84
ABC (60 mg)	$8.21	$10.94	$12.36
d4T (1 mg/ml)	$5.47	NR	$4.56
d4T (15 mg)	NR	$1.22	$1.44
3TC (10 mg/ml)	$2.43	$3.65	$2.06
LPV/r (80/20 mg/ml)	$17.33	$23.10	$11.81
LPV/r (100/25 mg)	NR	$12.77	$11.20
NVP (10 mg/ml)	$4.86	$6.08	$4.81
NVP (50 mg)	$4.10	$5.47	N/A
ZDV (10 mg/ml)	$5.47	$7.30	$4.82
ZDV (60 mg)	$4.56	$6.08	N/A
ZDV/3TC (60 mg/30 mg)	$4.56	$6.08	$5.60
ZDV/3TC/NVP (60 mg/30 mg/50 mg)	$6.38	$8.51	$8.28
d4T/3TC (6 mg/30 mg)	$2.74	$3.65	$3.79
d4T/3TC/NVP (6 mg/30 mg/50 mg)	$3.65	$4.86	$4.66
ABC/3TC (60 mg/30 mg)	$8.21	$10.94	$13.96

*Abbreviations*: ***ABC*** abacavir, ***d4T*** stavudine, ***3TC*** lamivudine, ***EFV*** efavirenz, ***LPV/r*** lopinavir/ritonavir, ***NVP*** nevirapine, ***ZDV*** zidovudine, ***mg*** milligrams, ***ml*** milliliter, ***NR*** not recommended, ***N/A*** not applicable (no cost estimate was available from the GPRM for this formulation).

A. Pediatric formulations in the WHO GPRM are based on costs for a 10 kg child. We have shown our calculations from Table 3 for weight bands 6–9.9 kg and 10–13.0 kg as a comparison.

**Table 8:** Comparison of adult formulation derived costs to the WHO Global Price Reporting Mechanism (GPRM) database

**Medication (strength)**	**Monthly costs for 35 + kg (from Table **3**)**	**Monthly costs from GPRM**
ABC (300 mg)	$15.81	$13.39
d4T (30 mg)	$1.82	$1.52
3TC (150 mg)	$2.43	$2.36
EFV (600 mg)	$4.56	$3.86
LPV/r (200/50 mg)	$31.62	$29.47
NVP (200 mg)	$3.04	$2.47
ZDV (300 mg)	$7.30	$6.98
ZDV/3TC (300 mg/150 mg)	$9.12	$7.86
ZDV/3TC/NVP (300 mg/150 mg/200 mg)	$11.55	$9.89
d4T/3TC (30 mg/150 mg)	$3.65	$2.99
d4T/3TC/NVP (30 mg/150 mg/200 mg)	$6.69	$4.76
ABC/3TC (600 mg/300 mg)	$17.72-$32.53	$22.56

*Abbreviations*: ***ABC*** abacavir, ***d4T*** stavudine, ***3TC*** lamivudine, ***EFV*** efavirenz, ***LPV/r*** lopinavir/ritonavir, ***NVP*** nevirapine, ***ZDV*** zidovudine, ***mg*** milligrams, ***ml*** milliliter, ***NR*** not recommended, ***N/A*** not applicable (no cost estimate was available from the GPRM for this formulation).

## Discussion

In this paper, we have provided a standardized methodology for estimating monthly pediatric ARV costs that could allow for greater comparability between future analyses of cost-effectiveness. Our approach incorporates changing dosing requirements as children grow, and accounts for a range of scenarios for the availability of pediatric ARV formulations in different countries and settings. To date, there have been no other published procedures for estimating pediatric ART costs by age and by drug regimen. Meyer-Rath *et al*. focused their analysis on calculating the average cost of HIV treatment care across at two pediatric care sites in South Africa [23]. They further estimated that approximately $25/month (or 40% of the total per-patient cost over the two years following treatment initiation) pertained to pediatric ART costs. Recommended pediatric regimens at these sites included d4T/3TC/LPV/r for children less than 3 years of age, and d4T/3TC/EFV for children greater than 3 years of age, with a median age of 4–6 years at initiation across the two sites. In our analysis, we found similar monthly costs for d4T/3TC/LPV/r, which ranged from $18.40-$21.47 for children ages 4–6 years old, depending on formulation assumptions. However, our monthly cost estimates for d4T/3TC/EFV ($7.64-$9.53) for children ages 4–6 years old, were significantly lower (Table 6). This discrepancy could be due to additional programmatic costs (cold chain storage, shipping costs, etc.) that may have also been included in the pediatric ART costs in the Meyer-Rath estimation.

In addition, the WHO has compiled average monthly costs for several pediatric and adult drug formulations in their GPRM database based on records of international transactions of HIV, tuberculosis and malaria products purchased in low- and middle-income countries [36], focused on 10-kg children. Here, our monthly drug costs for children in weight bands 6–9.9kg and 10–13.9 kg were fairly comparable or slightly higher for most formulations (Table 7). We did find, though, that our estimate of the liquid LPV/r formulation (80/20 mg/ml), which ranged from $17.33-$23.10 was almost double the cost reported in the WHO GPRM Database ($11.81). This discrepancy is likely due to the inherent differences in a ceiling price list (used in our analysis for unit cost derivation) and the actual transaction costs found in the GPRM. For example, despite the high cost of liquid LPV/r (80/20mg/ml), many countries and programs may have been able to negotiate its purchase at a lower cost than listed in the CHAI price list because of its key role in pediatric HIV treatment. In addition, the GPRM database shows that prices for LPV/r have fallen substantially over the past five years, from $16.11 to $11.81 per month, demonstrating the rapidly changing economic market for this drug. Our costing methodology described in this paper provides a more detailed and transparent estimation of ART costs over several ages, which will be useful in formulating pediatric ART recommendations in the future.

Our costing procedure has several limitations. First, we describe a step-by-step approach for calculating drug-related pediatric ART costs. These costs do not take into consideration equipment, personnel, overhead, shipping or opportunity costs that may also be involved in the delivery of ART to pediatric patients. We have also excluded the costs of establishing and maintaining a cold chain store needed for drugs that require refrigeration. The costs of these components are infrequently reported, but will likely inflate LPV/r liquid formulation costs by 5-50% depending on program level cold chain storage capabilities. Although we have chosen to exclude these variable costs from our analysis, the investments needed to establish a cold chain facility may be available at the program level and will be important considerations for program planners to include. In addition, because entire bottles of liquid formulation must be dispensed monthly, often exceeding the total needed quantity, there will likely be some wastage associated with the distribution of liquid drugs. Our current methodology excludes wastage costs, but these will need to be considered by program planners as well. Our costs estimates, however, will allow providers to incorporate drug-related pediatric ART costs into their existing budget and thus permit this methodology to apply to a variety of clinical settings. And finally, pediatric ART costs will likely change over time with the addition of new formulations of existing drugs or the development of new pediatric and adult medications. For example, LPV/r sprinkles and modifiable-dose ARV sachets are currently under development [37]. These formulations may improve tolerability and dosing flexibility, and also may also remove storage requirements that are a significant barrier to scale up of LPV/r use. A cost estimate has not yet been released for these formulations, but will be necessary to determine their cost effectiveness in the context of other available ARV options [37]. The methodology described here can be used as a framework for these and other drug developments in the future.

## Conclusions

The number of children living with HIV is growing each year, and will likely continue to grow until efforts to prevent perinatal HIV infection reach target goals [38]. Antiretroviral therapy has been shown to drastically reduce morbidity and mortality in children with HIV, yet questions about when to start treatment and what treatment to start remain key issues for policy makers [2,39]. In order to thoroughly examine these questions, one must be able to accurately assess the costs of pediatric antiretroviral therapy. We hope that the methodology outlined in this paper can be used as a standardized approach for budget administrators and cost-effectiveness analysts, who seek to increase pediatric ART availability in a constrained budget environment and that our detailed procedure will allow for more accurate comparison of pediatric drug costing across methodologies and evaluations. This costing procedure can be modified to reflect ARV prices and available formulations that are most representative for a range of programs and settings, and thus can aid budget analysts in developing more cost-effective recommendations for pediatric ART.

## Abbreviations

3TC: Lamivudine; ABC: Abacavir; ART: Antiretroviral therapy; ARV: Antiretroviral; CDC: Center for Disease Control; CHAI: Clinton Health Access Initiative; D4T: Stauvudine; EFV: Efavirenz; FDC: Fixed dose combination; GPRM: Global Price Reporting Mechanism; LPV/r: Lopinavir/ritonavir; MSF: Mèdecins Sans Frontières; NVP: Nevirapine; PEPFAR: The U.S. President’s Emergency Plan for AIDS Relief; WHO: World Health Organization; ZDV: Zidovudine.

## Competing interests

Dr Holmes was employed by Gilead Sciences from July 2006-January 2008 and was a stockholder during that time. All other authors declare they have no competing interests.

## Authors’ contributions

AC and KD developed the costing methodology and drafted the manuscript. SR developed the methodology for weight-to-age standards. SE, MP, CH and SR provided critical support to the costing procedure and revising the manuscript. All authors read and approved the final manuscript.

## Pre-publication history

The pre-publication history for this paper can be accessed here:

http://www.biomedcentral.com/1472-6963/14/201/prepub

## Supplementary Material

Additional file 1 Calculate monthly ART costs by regimen and age. Click here for file
